# A Quantitative Assessment of Pre-Operative MRI Reports in Glioma Patients: Report Metrics and IDH Prediction Ability

**DOI:** 10.3389/fonc.2020.600327

**Published:** 2021-01-29

**Authors:** Hang Cao, E. Zeynep Erson-Omay, Murat Günel, Jennifer Moliterno, Robert K. Fulbright

**Affiliations:** ^1^ Department of Neurosurgery, Xiangya Hospital, Central South University, Changsha, China; ^2^ Department of Neurosurgery, Yale School of Medicine, New Haven, CT, United States; ^3^ Department of Radiology and Biomedical Imaging, MRRC, Yale School of Medicine, New Haven, CT, United States

**Keywords:** glioma, magnetic resonance imaging, electronic health records, quality improvement, biomarkers

## Abstract

**Objectives:**

To measure the metrics of glioma pre-operative MRI reports and build IDH prediction models.

**Methods:**

Pre-operative MRI reports of 144 glioma patients in a single institution were collected retrospectively. Words were transformed to lowercase letters. White spaces, punctuations, and stop words were removed. Stemming was performed. A word cloud method applied to processed text matrix visualized language behavior. Spearman’s rank correlation assessed the correlation between the subjective descriptions of the enhancement pattern. The T1-contrast images associated with enhancement descriptions were selected. The keywords associated with IDH status were evaluated by χ2 value ranking. Random forest, k-nearest neighbors and Support Vector Machine algorithms were used to train models based on report features and age. All statistical analysis used two-tailed test with significance at p <.05.

**Results:**

Longer word counts occurred in reports of older patients, higher grade gliomas, and wild type IDH gliomas. We identified 30 glioma enhancement descriptions, eight of which were commonly used: peripheral, heterogeneous, irregular, nodular, thick, rim, large, and ring. Five of eight patterns were correlated. IDH mutant tumors were characterized by words related to normal, symmetric or negative findings. IDH wild type tumors were characterized words by related to pathological MR findings like enhancement, necrosis and FLAIR foci. An integrated KNN model based on report features and age demonstrated high-performance (AUC: 0.89, 95% CI: 0.88–0.90).

**Conclusion:**

Report length depended on age, glioma grade, and IDH status. Description of glioma enhancement was varied. Report descriptions differed for IDH wild and mutant gliomas. Report features can be used to predict glioma IDH status.

## Introduction

The pre-operative MRI scan of patients with glioma plays both a critical role in aiding diagnosis (1), the surgical approach, and also in serving as a baseline reference for future management. MR signal intensity data is easily available, and the MR scan data have been the subject of intense focus as researchers seek to analyze MR data in a qualitative and quantitative manner (2). Through advanced MR imaging method and radiomics analysis, the pre-operative genotype (3), immune phenotype (4), and prognosis prediction (5) become possible. These findings based on image data greatly expand the clinician’s vision on glioma pathogenesis and treatment strategies.

The value and properties of another very important part of the pre-operative MRI data, the radiology report, have not been so well appreciated. Previous studies have put efforts into certain aspects of reports including tumor volume estimation (6) and treatment response evaluation (7), but the deconstruction and analysis of the full text are still lacking. In clinical practice, the qualitative and quantitative comments contained in MRI report remain the major point of reference for many diagnostic and treatment decisions. The major difficulties of using MRI reports data could be divided into three parts. First, the text reports are of non-structured data, which is largely due to the natural language used in reports. The practice years, personal habit, and language background may change the focus and sentence order of text reports. Second, the heterogeneous MRI appearance of glioma results in a variable subjective description of imaging findings. Third, the general properties of disease-specific reports have not been established using quantitative analyses designed to analyze natural language. Interestingly, the above-mentioned difficulties could also be advantages to study radiology reports. Unlike the neural network models and many machine learning models, the models based on natural language-based reports have inherent interpretability. Also, the reports as unchangeable medical record could better reflect real-world judgement and largely diminish the possible retrospective bias.

Our study objectives were to characterize glioma pre-operative MRI reports and to assess the IDH prediction ability of report features. We collected the pre-operative MRI reports on glioma patients and analyzed them with automatic natural language processing algorithm. The processed and tokenized report features were then used to build IDH prediction models. The success rate of the prediction algorithm was calculated using the IDH status identified in the cases with whole exome sequencing or by targeted sequencing.

## Materials and Methods

### Patients and Reports

We performed a retrospective review of 144 consecutive cases between 2011 and 2018. For inclusion, patients who consented for clinical research had a confirmed pathological diagnosis of astrocytic or oligodendroglial tumor at our institution and underwent routine pre-operative MRI brain scan at the same institution. Full-length text reports of pre-operative MRI scans were collected from the picture archival and communication system. If the patient underwent more than one pre-operative MRI, the first report was included to prevent retrospective bias.

### MRI Protocol

See **Supplementary File 1**.

### Histology Subtypes and IDH Status

Surgical tumor samples underwent post-operative histology assessment, in which the integrated diagnosis was made based on the histological features, the initial IDH R132H status (provided by immunohistochemical IHC method), and MGMT promoter status (determined by PCR spell out method). The subsequent IDH mutation status was determined by targeted or whole exome sequencing.

### The Extraction of Meta Data From Text Reports

MRI text reports included study indication, clinical history, comparison studies, imaging technique, findings, impression, dictating radiologist, and co-signing radiologist. To minimize the experience related bias and privacy exposure, only the findings section was used for building the text report or corpus. Meta data of the text reports was extracted through manual or automatic methods. Manual extraction was performed for scan date, dictating radiologist and co-signing radiologist. Automatic extraction was performed for word count and sentence count using R package quanteda (version 2.0.1) (8).

### Detection and Correction of Misspellings

Misspellings were identified and corrected *via* R package hunspell (version 3.0) (9) with plug-in medical term dictionary and by manually checking. The corrected text matrix was used for the next analysis, below.

### Processing and Visualization of the Text Reports

Reports underwent lowercase transformation, number removal, punctuation removal, stopword removal and stemming processing to build sparse document-feature matrix (DFM) *via* R package quanteda (version 2.0.1). The DFM was used to generate a word cloud with the top 100 frequent tokens. A token is the word after the above processing was performed.

### Detection of Abbreviations

The R package clean-NLP (version 3.0.0) with udpipe annotation algorithm was used for abbreviation identification. After ranking the annotated tokens by length, tokens with less than 10 letters were manually checked. For potential abbreviations, the original sentence was inspected to confirm the full word and original meaning.

### Frequency and Redundancy Analysis of the Subjective Description of Enhancement Patterns

We manually labeled sentences containing the enhancement description from each report. Enhancement pattern modifiers were extracted from sentences. The frequency of enhancement pattern modifiers was summarized. Modifiers used in at least five or more reports underwent spearman correlation test. We collected T1-contrast images corresponding to enhancement patterns described in reports.

### Analysis of Keywords Associated With IDH Status

Keywords associated with IDH status were analyzed based on the extracted document feature matrix (DFM). Keyword preference was evaluated by the keyness score, which equals the calculated chi-square value (Yates correction applied). The positive or negative keyness score sign was based on the relationship between observed value and expected value of target token relative to the IDH-MT corpus (**Supplementary File 1**). We summarized keywords with the 10 largest absolute keyness scores in the positive and negative sign groups. Since word counts with very low-frequency could also have high absolute chi-square value by coincidence, words occurring no more than five times were labeled. The p-values of all chi-square tests (Yates correction applied) were given.

### Establishment of IDH Status Prediction Model Based on Pre-Operative MRI Reports

Report-based glioma IDH prediction models were built *via* Orange software datamining tool (10) (version 3.25). Tokens were used as input features for model training. Samples were separated into 70% training set (97 patients) and 30% test set (42 patients) for 100 times. Machine learning methods included support vector machine, random forest, and k-nearest neighbors. Machine learning model parameters are provided in **Supplementary File 1**. Average performance of the models was evaluated by AUC, sensitivity and specificity, by setting the IDH mutant type as target class. Since age is a predictive factor for IDH status, we generated age logistic regression model and integrated machine learning models, using age and report features. The threshold of 0.5 (<0.5, IDH wild type ≥0.5, IDH mutant type.) was selected for all models.

### Statistical Analysis

The normality of continuous variables was first assessed by the Kolmogorov–Smirnov test. Due to insignificant results, non-parametric statistics tests were used to compare group differences. Pearson chi-square test, Fisher’s exact test and likelihood-ratio test were used for categorical variables (sex, histology subtype, WHO grade, IDH, and MGMT status) according to expected count range and contingency table type. Statistical tests were performed with SPSS software (version 22.0; IBM). For model performance evaluation, MedCalc (version 19.0.7; MedCalc Software) was used to assess AUC, sensitivity, specificity, and to compare ROC curves.

## Results

### Patient Characteristics

The characteristics of the 144 patients (84 men, 60 women) for which the median age and interquartile range were 59 years and 19 years, respectively are summarized in **Table 1**. Differences were found in age, initial histology reading, WHO grade, MGMT status between IDH mutant and wild type groups (P <.001). IDH wild type tumors were in older patients and were more commonly Grade IV tumors. IDH mutant tumors were more likely to be methylated with respect to MGMT. The inconsistency of diagnosis of oligodendroglioma and IDH status based on initial histology reading *versus* subsequent whole exome sequencing was found in one patient labeled as having anaplastic oligodendroglioma. There was an imbalance between IDH subtype distributions (31 IDH mutant *vs* 107 wild type patients).

**Table 1:** Clinical characteristics of the study samples.

Item		Study cohort (n = 144)	IDH mutant glioma (n = 31)	IDH wild type glioma (n = 107)	p value
Age					
	Median (Q1**–**Q3)	59 (50**–**69)	39 (33**–**51)	63 (54**–**71)	<.001
Sex					
	Women	60	16	42	0.22
	Men	84	15	65	
Histology					
	Diffuse astrocytoma	12	10	2	<.001
	Oliogoastrocytoma	2	2	0	
	Oligodendroglioma	8	7	0	
	Anaplastic astrocytoma	8	4	3	
	Anaplastic oligoastrocytoma	2	1	1	
	Anaplastic oligodendroglioma	6	5	1	
	Diffuse midline glioma, H3 K27M-mutant	2	0	2	
	Glioblastoma	104	2	98	
WHO grade					
	Grade II	21	19	2	<.001
	Grade III	16	10	5	
	Grade IV	106	2	100	
IDH status					
	Wild type	107	0	107	NA
	Mutant type	31	31	0	
	NA	6	0	0	
MGMT status					
	Methylated	70	26	42	<.001
	Unmethylated	68	3	62	
	NA	6	2	3	
Reports meta feature: Radiologists					
	Report radiologist (persons)	47	17	38	NA
	Co-sign radiologist (persons)	22	14	20	
	Total radiologist (persons)	68	31	57	

### General Characteristics of Reports

A total of 144 MRI reports signed by academic neuroradiologists were included in this study. The radiologist numbers are summarized in **Table 1**. All reports were generated using microphones with voice recognition software. A dictation template was used that had headings of study, date, study indication, and clinical history, whether comparison studies were available, imaging technique, findings, and impression. The date was auto-populated. There were sentences included in the template about normal structures that could be used or changed. Findings were dictated by the radiologists into the appropriate fields. The findings part of the reports started with the lesion location and size description, followed by routine conventional sequence (T1, T1-contrast, T2, FLAIR) appearance and optional advanced sequence (DWI, SWI, PWI, MRS) characteristics. The report findings ended with negative findings (findings not present) and assessment of normal structures.

One hundred twenty of one hundred forty-four reports included measurements of the glioma. In 13 of 144 reports, the statement that the study was limited was found at the beginning (10), middle (two) or end (one) of the reports. The evaluation of negative findings included transtentorial herniation, hydrocephalus, ventricle asymmetry, midline shift, global parenchymal volume loss, acute infarct, and acute intracranial hemorrhage. Evaluation of normal structure included extra axial space, ventricular system, osseous structure, orbits, and paranasal sinuses. One hundred thirty-three reports that used free-text description in evaluation of normal structures. One misspelled word was found in all 144 reports (“tthe”, should be “the”). This misspelling was corrected before further analysis.

Processing of the 144 MRI reports generated a text corpus for each report, resulting in 144 processed corpora (1,476 sentences, 21,357 words). The word and sentence count characteristics were summarized in **Table 2**. Word counts were longer in older patients (p < 0.05). In all samples, patients with WHO grade II glioma were younger (p < 0.001) and had reports with lower word counts (p = 0.01) when compared with patients with WHO grade III and IV gliomas. In cases with an available IDH status, patients with an IDH mutant glioma were younger (p < 0.001) and had lower word counts in reports (p = 0.027) when compared with IDH wild type gliomas. Patients with WHO grade III glioma were younger (p = 0.006) but had similar word counts (p = 0.873) when compared with patients with WHO grade IV glioma. The largest word count interquartile range was found in WHO grade III glioma reports (median and interquartile range, 161.5 and 85.5). No difference was found in sentence count according to age, gender. WHO grade, IDH status, or MGMT status. The median sentence count of 10 was found in all groups.

**Table 2:** The word and sentence count of the study reports.

Item	Word count	p value	Sentence count	p value
	Median (Q1–Q3)		Median (Q1–Q3)	
Total study samples (n = 144)	144.5 (111.5–176.5)	NA	10 (8–12)	NA
Age (n = 144)				
≤40 years (n = 23)	113 (98–173)	0.02*	10 (8–12)	0.42
>40 and ≤60 years (n = 54)	140.5 (105–168)		10 (8–11)	
>60 years (n = 67)	155 (121–185)		10 (8–12)	
Sex (n = 144)				
Woman (n = 60)	147.5 (120.5–181.5)	0.20	10 (9–12)	0.17
Man (n = 84)	138 (107.5–172)		10 (8–11)	
WHO grade (n = 144)				
Grade II (n = 22)	110 (103–148)	0.04*	10 (9–12)	0.91
Grade III (n = 16)	161.5 (104–189.5)		10 (8.5–12.5)	
Grade IV (n = 106)	146.5 (120–178)		10 (8–12)	
IDH status (n = 138)				
Wild type (n = 107)	147 (120–181)	0.03*	10 (8–12)	0.74
Mutant type (n = 31)	113 (102–168)		10 (9–12)	
MGMT status (n = 138)				
Methylated (n = 70)	137 (107–177)	0.60	10 (8–12)	0.96
Unmethylated (n = 68)	146.5 (118–178.5)		10 (8–12)	

*Significant.

**Figure 1:**
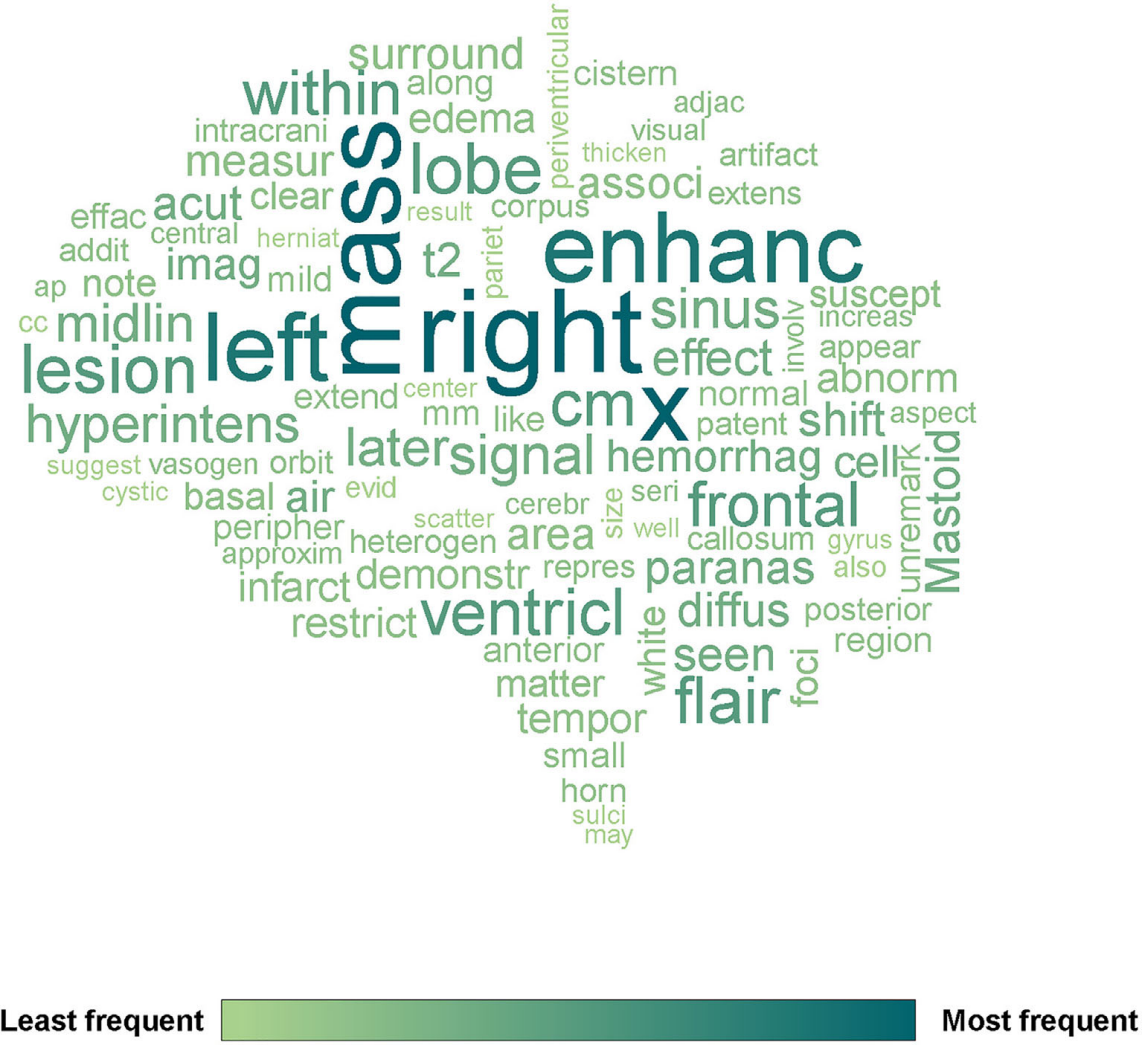
The wordcloud of 144 pre-operative glioma MRI reports generated by R package quanteda. The top 100 tokens were selected. X means tumor measurement.

The top 100 tokens (processed words) are shown in **Figure 1**. The most frequent words were related to body side, tumor size measurement (“x”), anatomical location, enhancement and other MR signal descriptions. The brain ventricle was also frequently mentioned in mass effect and infiltrated region.

### Abbreviations

A total of 27 abbreviations were found in all reports. The abbreviation frequency, full word and category are summarized in **Table 3**. The abbreviations used could be classified into six categories including MRI term, CT term, anatomy, pathology, chemistry, and direction of imaging plane. The word transverse was found to have the most variant abbreviation presentations, which included TR, TRV, TV.

**Table 3:** The abbreviations used in the MRI reports.

Abbreviations	Full word	Category	Count
FLAIR	Fluid-attenuated inversion recovery	MRI term	170
T2	T2 relaxation	MRI term	111
AP	Anterior-posterior	Direction	56
CC	Craniocaudal	Direction	47
T1	T1 relaxation	MRI term	28
TR	Transverse	Direction	21
TRV	Transverse	Direction	20
CT	Computed tomography	CT term	13
TV	Transverse	Direction	7
CSF	Cerebrospinal fluid	Anatomy	6
A2	Second segment of the anterior cerebral artery	Anatomy	4
M1	First segment of the middle cerebral artery	Anatomy	3
M2	Second segment of the middle cerebral artery	Anatomy	3
MCA	Middle cerebral artery	Anatomy	3
SWI	Susceptibility weighted imaging	MRI term	3
DTI	Diffusion Tensor Imaging	MRI term	2
DWI	Diffusion weighted imaging	MRI term	2
C2	Second cervical vertebra	Anatomy	1
ADC	Apparent diffusion coefficient	MRI term	1
C1	First cervical vertebra	Anatomy	1
CTA	Computed tomography angiography	CT term	1
DVA	Developmental venous anomaly	Pathology	1
MPRAGE	Magnetization-prepared rapid gradient-echo	MRI term	1
NAA	N-acetylaspartate	Chemistry	1
rCBV	Relative cerebral blood volume	MRI term	1
SI	Superior-inferior	Direction	1
VIII	Eight	Anatomy	1

### Subjective Description of Enhancement Patterns

**Figure 2:**
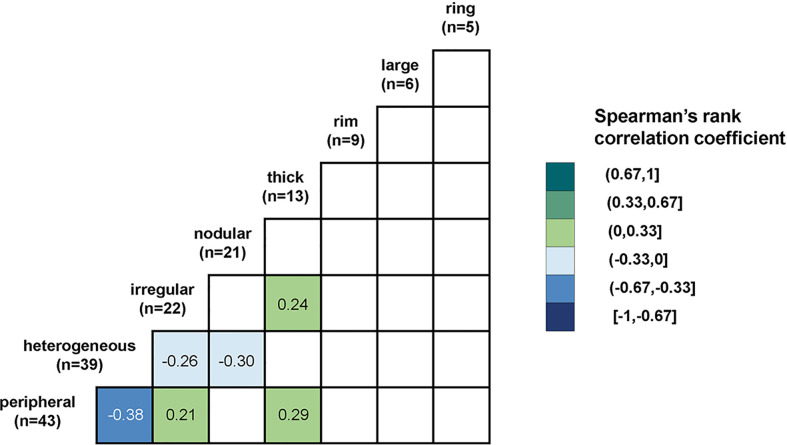
The correlations between major descriptions of enhancement patterns.

**Figure 3:**
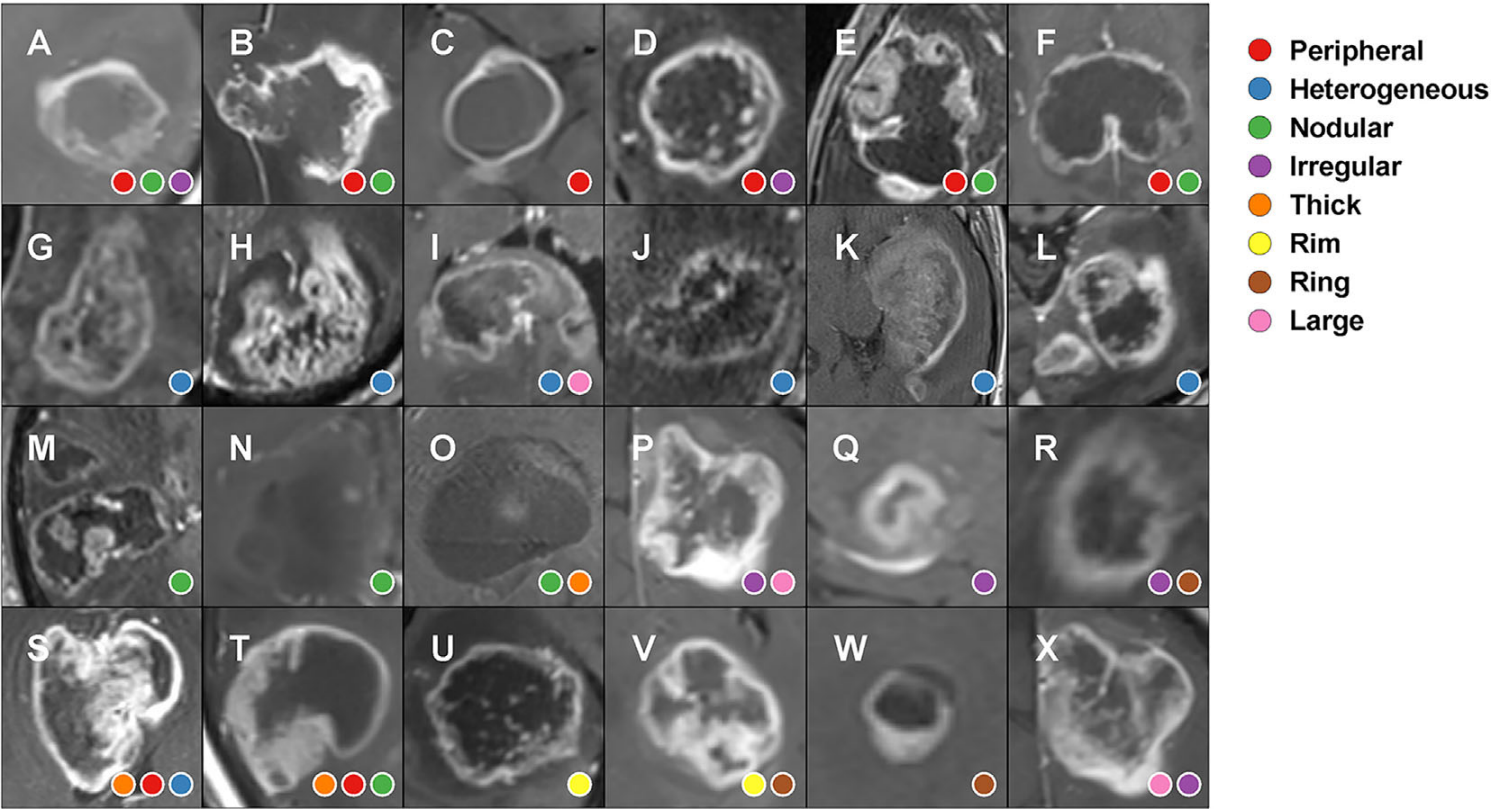
Glioma enhancement patterns and how radiologists described them in pre-operative MRI reports. **(A–F)** peripheral enhancement; **(G–L)** heterogeneous enhancement; **(M–O)** nodular enhancement; **(P–R)** irregular enhancement; **(S, T)** thick enhancement; **(U, V)** rim enhancement; **(W)** ring enhancement; **(X)** large enhancement.

There were 112 of 144 reports (77.8%) that included a subjective description of enhancement. Thirty subjective descriptions were identified and are summarized in **Supplementary File 1**. Eight of 30 (26.7%) subjective descriptions that occurred in at least five reports were taken to be major enhancement patterns. These included peripheral, heterogeneous, irregular, nodular, thick, rim, large, and ring. The frequency and Spearman’s rank correlation coefficient of major enhancement patterns are shown in **Figure 2** (the coefficient was only shown when p <.05). The largest absolute correlation coefficient was found between peripheral and heterogeneous enhancement (r = −0.38, p <.001). Thick enhancement demonstrated positive correlation with peripheral enhancement description (r = 0.29, p = .002). Nodular enhancement showed negative correlation with heterogeneous enhancement description (r = −0.30, p = .001). Thick enhancement was positively correlated with irregular enhancement description (r = 0.24, p = .01). Interestingly, irregular enhancement showed both positive correlation with peripheral enhancement (r = 0.21, p = .03) and negative correlation with heterogeneous enhancement description (r = −0.26, p = .004). The representative T1-contrast images corresponding to the subjective descriptions of the major enhancement patterns are in **Figure 3**.

### Keywords Associated With IDH Status

**Figure 4:**
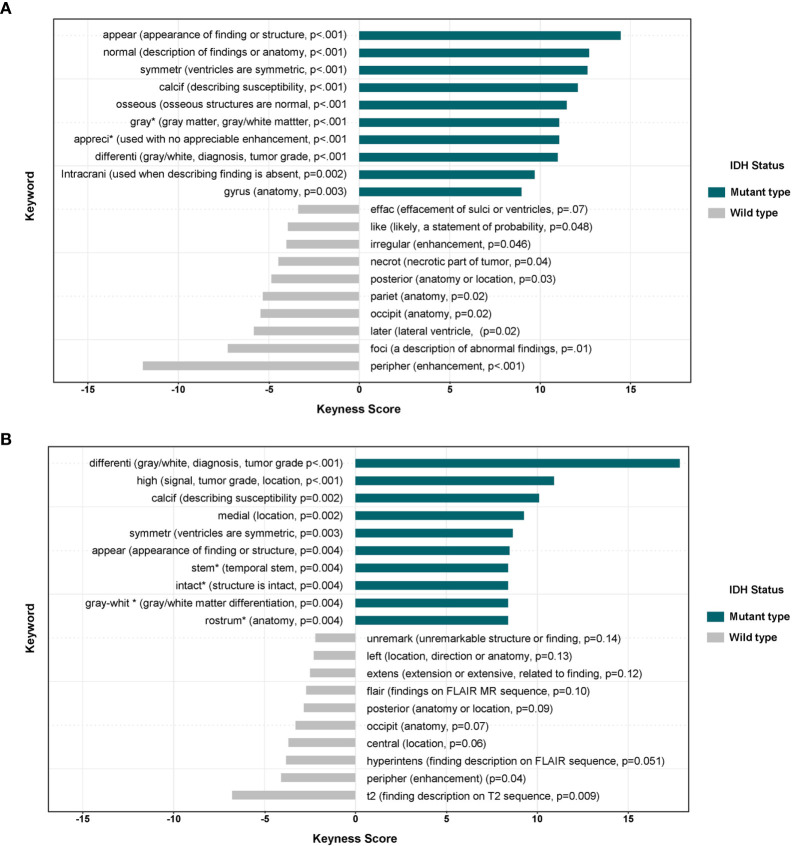
**(A)** The differential associations of keywords in MRI reports in IDH mutant and wild type gliomas (grades II**–**IV, n = 138). The positive keyness score was correlated with IDH-mutant type glioma. *Infrequent description in reports (n < 5 reports). **(B)** The differential associations of keywords in MRI reports in IDH mutant and wild type gliomas (grades III**–**IV, n = 117). The positive keyness score was correlated with IDH-mutant type glioma. *Infrequent description in reports (n < 5 reports).

The keywords occurring differentially based on in IDH mutant and wild type gliomas are shown in **Figure 4A** (all grade samples) and **Figure 4B** (Grade III and IV samples). IDH mutant type gliomas were characterized by keyness values with positive signs. Keywords associated with IDH mutant tumors were associated with normal findings, symmetric ventricles, normal gray matter, no appreciable enhancement, no intracranial findings, normal appearance, or intact structures. IDH wild type gliomas were characterized with keyness values with negative signs. Keywords associated with IDH wild type gliomas were associated with effacement, irregular enhancement or shape, necrosis, foci of abnormal findings, peripheral enhancement, extension of signal, FLAIR findings, T2 findings, and peripheral enhancement.

### IDH Status Prediction Based on Pre-Operative Reports

The overall test set results of model performance are summarized in **Table 4**. The integrated KNN model which used both age and report feature achieved the highest test set AUC (0.89, 95% CI: 0.88–0.90). The age only logistic regression model achieved the second highest test set AUC (0.87, 95% CI: 0.86–0.88). The pairwise comparison of ROC curves between integrated model (age and report feature) and age only model showed statistical difference (p < 0.001). The integrated KNN model showed higher sensitivity than age only logistic regression model (Target class: IDH mutant type. 58.9 *vs* 21.3%). For report feature models, SVM model showed highest AUC (0.75, 95% CI: 0.73–0.76). All models showed high specificity (>85%) and low sensitivity (<60%).

**Table 4:** The performance of the IDH prediction model from 100 test sets.

Feature group	Modeling method	AUC	95% CI	Sensitivity	95% CI	Specificity	95% CI
Age	Logistic Regression	0.87	0.86–0.88	21.3% (192/900)	18.7–24.2%	96.1% (3170/3300)	95.3–96.7%
Report text feature	KNN	0.65	0.63–0.66	26.4% (238/900)	23.6–29.5%	88.4% (2916/3300)	87.2–89.4%
	SVM	0.75	0.73–0.76	16.4% (148/900)	14.1–19.0%	97.2% (3209/3300)	96.6–97.8%
	RF	0.65	0.64–0.67	9.4% (85/900)	7.6–11.5%	97.7% (3225/3300)	97.2–98.2%
Report text feature + Age	KNN	0.89	0.88–0.90	58.9% (530/900)	55.6–62.1%	90.6% (2988/3300)	89.5–91.5%
	SVM	0.77	0.76–0.79	22.3% (201/900)	19.7–25.2%	96.6% (3188/3300)	95.9–97.2%
	RF	0.73	0.72–0.75	14.8% (133/900)	12.5–17.3%	97.2% (3207/3300)	96.6–97.7%

## Discussion

The pre-operative MRI reports of gliomas read by neuroradiologists in an academic institution were analyzed to provide report metrics and IDH prediction ability. Longer word counts were found in older patients and in patients with high grade (WHO grades III and IV) gliomas and wild type IDH tumors. The patients with WHO grade III glioma were younger and showed similar word counts compared with patients with WHO grade IV glioma. We identified 30 different subjective enhancement descriptions, in which eight descriptions were commonly used in at least five reports. These included “peripheral”, “heterogeneous”, “irregular”, “nodular”, “thick”, “rim”, “large”, and “ring”. Five of these eight descriptions were correlated (p < 0.05). Reports in IDH mutant gliomas used words like normal, symmetric, and intact. Reports in IDH wild type gliomas used words like enhancement, necrosis, and abnormal signal intensity on FLAIR sequences. The prediction model generated by age and report features *via* KNN method showed better performance than age only model (AUC, 0.89 *vs* 0.87. IDH mutant detection sensitivity, 58.9 *vs* 21.3%).

During the past decades, efforts have been made to improve brain tumor MRI reporting. The major approaches included a focus on structured reporting and general quality. For structured reporting, Mamlouk et al (11). in 2018 introduced a series of neuroradiology structured templates that contained key points to report based on specific indications. In the report template for head and neck cancer, the findings part of the report required the radiologist to report tumor and nodal characteristics. Apart from pre-designed options, the template still has the free-text description part. In our reports, we found the structured system resulted in nearly similar sentence count for all reports. Andrea et al (12). in 2018 compared the reporting with an expert designed tumor MRI report template with free-text reporting. Findings were more completely reported when using a template. Our reports covered the majority of template fields suggested by Andrea et al., but the lesion size was usually measured on single sequence instead of both T1-contrast and FLAIR. Our reports contained more detailed tumor components’ (enhancing tumor, necrosis, edema) pattern description, which have been found to correlate with glioma molecular subtypes.

For general quality, we found our reports were satisfactory as there were few misspellings, and abbreviations were easy to understand. However, the enhancement patterns were described in a variable way and may not be fully objective. For example, the word “irregular” was found preferably used with “peripheral” and not used with “heterogeneous”. This finding suggested there is the potential risk of biased description caused by language habits. The variable appearance of glioma enhancement on MR leads to a multiplicity of language-based descriptions, in part due to using synonyms or related words to describe a similar imaging feature (**Figure 3**). The varied descriptions seen in **Figure 3** suggest that a more standardized manner of describing contrast enhancement might be useful to the field.

Among various genomic alternations of glioma, the IDH mutation has attracted attention due to its diagnosis and prognosis prediction value. MRI features including enhancement ratio (13), MRS 2HG signal (14), radiomics features (15), and deep learning features (16) have been used to build pre-operative prediction models. However, processing procedures like drawing tumor region or additional scanning are required to generate such features for prediction models. In our study, the language features of report findings were directly extracted without image processing procedures. The keyword analysis suggested IDH mutation status was associated with different descriptions in reports. The keywords differentiating mutant and wild types were mainly about negative imaging findings, enhancement, necrosis, edema, and anatomy. When used with age information, the report features generated a satisfactory prediction model. This type of model could be directly implemented in the daily workflow without requiring extra imaging analysis. Additional research could explore other features that radiology reports might have in providing information about brain tumors.

Our study has several limitations. First, the study cohort was retrospectively collected from a single institution. Second, the imbalance of IDH status distribution in the study cohort might be a result of referral bias of our institution. This imbalance could affect the specific keyword analysis and prediction model training. A more balanced and larger cohort would be helpful for further research. Third, the selection procedure of representative T1 contrast images could contain subjective bias since all imaging sequences were not analyzed. Finally, all of the radiologists who signed the final report were academic neuroradiologists. Our findings might not be applicable to a general radiology practice.

MRI reports were longer in older patients, high grade gliomas, and IDH wild type gliomas. Patients with WHO grade III glioma were younger but had similar word counts compared to patients with WHO grade IV glioma. The descriptions of glioma enhancement were variable. Five of the most common descriptions of enhancement patterns were correlated. Keyword analysis of radiology reports demonstrated different descriptions were used for IDH wild and IDH mutant gliomas. Text features of reports were used to build a model that could predict glioma IDH status.

## Data Availability Statement

The original contributions presented in the study are included in the article/**Supplementary Material**; further inquiries can be directed to the corresponding author.

## Ethics Statement

The studies involving human participants were reviewed and approved by the Yale Human Investigative Committee, Biomedical Board, Yale University School of Medicine. The patients/participants provided their written informed consent to participate in this study.

## Author Contributions

RF’s contributor roles include conceptualization (lead), development of the methodology (lead), supervision (lead), and writing, reviewing, and editing of the manuscript (lead). HC’s contributor roles include data curation (lead), formal analysis (Lead), development of the methodology (lead), visualization (lead), and writing of the original draft (lead). EE-O’s contributor roles include supervision (supporting), and writing, reviewing, and editing the manuscript (supporting). MG’s contributor role includes supervision (supporting). JM’s contributor role includes supervision (supporting). All authors contributed to the article and approved the submitted version.

## Conflict of Interest

The authors declare that the research was conducted in the absence of any commercial or financial relationships that could be construed as a potential conflict of interest.

## Abbreviations

2HG, 2-Hydroxyglutarate; GBM, Glioblastoma; IDH, Isocitrate dehydrogenase; KNN, K nearest neighbors; MRS, Magnetic resonance spectroscopy; MGMT, O6-methylguanine-DNA methyl-transferase; PWI, Perfusion-weighted imaging.
