# Supplementary material for: A Quantitative Assessment of Pre-Operative MRI Reports in Glioma Patients: Report Metrics and IDH Prediction Ability
Source: Front Oncol. 2021 Jan 29;10:600327. doi: 10.3389/fonc.2020.600327 (PMC7879978; doi:10.3389/fonc.2020.600327)
Supplement: Supplementary file 1 [file DataSheet_1.docx]

**Supplementary file 1**

1. Machine learning parameters.

Software: Orange data mining, version 3.25

Learner #1: KNN

Number of neighbours: 5

Metric: Euclidean

Weight: Distance

Learner #2: SVM

SVM type: SVM, C=1.0, ε=0.1

Kernel: RBF, exp(-auto|x-y|²)

Numerical tolerance: 0.001

Iteration limt: 100

Learner #3: Random Forest

Number of trees: 10

Maximal number of considered features: unlimited

Replicable training: No

Maximal tree depth: unlimited

Stop splitting nodes with maximum instances: 5

Learner #4: Logistic Regression

Regularization: Ridge (L2), C=1

1. 30 subjective descriptions

Asymmetric

Avidly

Central

Circumscribed

Discontinuous

Few

Heterogeneously

Homogenously

Ill-defined

Internal

Irregular

Large

Marginal

Mild

Minimal

Moderately

Multiple

Mural

Nodular

Patchy

Peripheral

Predominantly

Rim

Ring

Satellite

Scattered

Serpiginous

Small

Subtle

Thick

1. The keyness score calculation.

In this study, we used the function textstat_keyness offered by R package quanteda to calculate the keyness score. The function details can be found on <https://quanteda.io/reference/textstat_keyness.html>.

Example:

A 2×2 contingency table was established based on target token and all other tokens’ **observed frequency** in IDH-MT and IDH-WT report corpus.

Target token All other tokens Total tokens

IDH-MT A C G

IDH-WT B D H

Total E F I

We can calculate the expected value in each cell via the formula:

**Expected value** = Row total * Column total / Number of total tokens in the corpus

The expected value of each cell would be:

Target token All other tokens Total tokens

IDH-MT E*G/I F*G/I G

IDH-WT E*H/I F*H/I H

Total E F I

Then, the chi-square value could be calculated via formula:


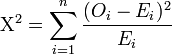
 or


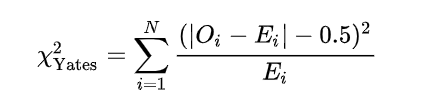


(when at least one cell of the table has an expected value smaller than 5, Yates's correction is used)

The absolute keyness score equals to the calculated chi-square value. The positive or negative sign is based on the relationship between observed value and expected value of target token relative to the IDH-MT corpus: When observed value A > expected value E*G/I, the keyness sign is positive. When observed value A < expected value E*G/I, the keyness sign is negative.

1. MRI Examination Protocol.

The MRI studies were performed on multiple 1.5T and 3.0T scanners (Magnetom Avanto; Magnetom Espree; Magnetom Symphony; Magnetom Triotim; Magnetom Skyra; Magnetom Verio; Magnetom Vida; Siemens. Signa Excite; GE Medical Systems). The routine MRI examination sequences included T1, T1-contrast, T2, FLAIR, DWI, PWI, SWI and optional MRS. The T1 scan slice thickness is 0.9-5.0 mm. The contrast agents were gadoterate meglumine and gadobutrol.
